# Usefulness of Hounsfield Units and the Serum Neutrophil-to-Lymphocyte Ratio as Prognostic Factors in Patients with Breast Cancer

**DOI:** 10.3390/cancers14143322

**Published:** 2022-07-07

**Authors:** Seok Hahn, Kwang-Min Kim, Min-Ju Kim, Hyang-Suk Choi, Hany Noh, In-Jeong Cho, Seung-Taek Lim, Jong-In Lee, Airi Han

**Affiliations:** 1 Department of Radiology, Inje University College of Medicine, Haeundae Paik Hospital, Busan 48108, Korea; h00367@paik.ac.kr; 2 Department of Surgery, Yonsei University Wonju College of Medicine, Wonju 26426, Korea; lukelike@yonsei.ac.kr (K.-M.K.); hamj@yonsei.ac.kr (M.-J.K.); hyangschoi@yonsei.ac.kr (H.-S.C.); withim@yonsei.ac.kr (H.N.); dite28@yonsei.ac.kr (I.-J.C.); 3 Department of Oncology, Yonsei University Wonju College of Medicine, Wonju 26426, Korea; darksgtlim@yonsei.ac.kr (S.-T.L.); oncohem@yonsei.ac.kr (J.-I.L.)

**Keywords:** breast neoplasm, vascularity, immunity, survival

## Abstract

**Simple Summary:**

Tumor vascularity and immune disturbances are hallmarks of cancer. Targeting agents against them have shown successful results. However, these agents were efficacious regardless of the presence of potential biomarkers. The need to understand this non-specific efficacy led us to focus on the crosstalk between them. We confirmed that each was an independent survival factor by utilizing the tumor-to-aorta ratio (TAR) of Hounsfield units on contrast-enhanced computed tomography and the serum neutrophil-to-lymphocyte ratio (NLR). We found that the survival disadvantage of TAR and NLR manifested only when the other factor was also unfavorable. Finally, we dichotomized patients into two groups, patients with unfavorable features of both TAR and NLR and others without, showing the survival disadvantage of the former group with statistical significance. We believe that this study provides a precise understanding of the crosstalk, with clinical data, promoting a virtual cycle of research from the bed to bench and vice versa.

**Abstract:**

Breast cancer is a leading cause of death worldwide. Tumor vascularity and immune disturbances are hallmarks of cancer. This study aimed to investigate the reciprocal effect of tumor vascularity, assessed by the tumor-to-aorta ratio (TAR) of Hounsfield units (HU) on computed tomography (CT), and host immunity, represented by the serum neutrophil-to-lymphocyte ratio (NLR) from peripheral, complete blood cell counts and its impact on patient survival. Female patients with breast cancer who received primary treatment between 2003 and 2018 at Wonju Severance Hospital, Korea, were included. The final cohort included 740 patients with a mean age of 54.3 ± 11.3 (22–89) years. The TAR was 0.347 ± 0.108 (range, 0.062–1.114) and the NLR was 2.29 ± 1.53 (0.61–10.47). The cut-off value for the TAR and NLR were 0.27 and 1.61, respectively. The patients with a TAR > 0.27 showed a poor recurrence free-interval (RFI) only when their NLR was larger than 1.61, and vice versa. The patients showed worse RFI when they had both high TAR and NLR. Our results suggest a dynamic reciprocal communication between tumor vascularity and systemic immunity.

## 1. Introduction

Breast cancer is one of the leading causes of death worldwide [1]. Tumor vascularity and immune disturbance are key hallmarks of cancer and are related to patient survival [2]. Tumor vascularity plays a fundamental role in promoting growth, invasion, and metastasis in various cancers, including breast cancer [3]. Thus, the evaluation of tumor vasculature is critical as it serves as the first step to identifying strong candidates for treatments, targeting tumor angiogenesis. Clinical imaging was proposed as a diagnostic modality to evaluate tumor vasculature, because of its non-invasiveness and potential to provide repetitive assessments of angiogenesis [4,5]. Increased tumor vascularity, assessed using computed tomography (CT) attenuation, has shown a significant correlation with patient survival [4,5]. The imaging techniques can also provide extractable information about the tumor, such as the underlying histopathology, the genetic makeup of the tumor, and the tumor microenvironment [6]. Interestingly, the most heavily represented category in radiogenomics involves the immune regulatory pathways [6]. Meanwhile, immune disturbance helps the cancer cells avoid the host’s destructive immunity, and prolonged inflammation has been known to be a hallmark of cancer [7]. The serum neutrophil-to-lymphocyte ratio (NLR) is known to impact the survival of patients with breast cancer [8,9,10]. While most studies reported an inverse relationship between the NLR and patients’ survival, not all of the patients in each subgroup shared the same results. In particular, this was observed in the patients who underwent neoadjuvant treatment and the patients with triple-negative breast cancer [8,9,10]. Intriguingly, increasing data suggest vascular-immune crosstalk, which enables dynamic reciprocal communication between these two factors [6,11,12,13]. This study aimed to evaluate the reciprocal influence of tumor vascularity, assessed by Hounsfield units (HU), on patients’ CT scans and hosts’ immune systems represented by the NLR and its impact on survival.

## 2. Materials and Methods

### 2.1. Study Cohort

This retrospective cohort study enrolled female patients diagnosed with primary stage I-III breast cancer between January 2003 and December 2018 who completed all the phases of planned treatment at Wonju Severance Hospital, Yonsei University, Wonju, Korea. Patients were eligible if they had stage I–III breast cancer and completed their planned systemic and local treatments. Patients were excluded if they had any of the following conditions: incomplete clinicopathologic, laboratory, or imaging data at the presentation of primary breast cancer; known stage IV disease; or conditions that can influence the NLR [8,9,10]. All of the records were coded by an independent data monitoring body and maintained by a neutral person (I-J CHO), who was blinded to the analysis. The survival analysis was based on two databases: Wonju Severance Hospital and the Korean National Cancer Registry.

### 2.2. Data Collection: Clinicopathological, Laboratory, Radiologic, and Survival Data

#### 2.2.1. Clinicopathological and Laboratory Data

The data regarding the subjects’ medical history, age, tumor size, lymph node status, estrogen receptor (ER) and progesterone receptor (PR) status, human epidermal receptor 2 (HER2) overexpression status, and laboratory data (complete blood cell count and differential white blood cell count) were collected.

The ER, PR, and HER2 statuses were mainly extracted from pathology reports and defined based on the American Society of Clinical Oncology/College of American Pathologists (ASCO/CAP) 2010 and 2013 guidelines [14,15]. The NLR was calculated as absolute serum neutrophil count/absolute serum lymphocyte count.

#### 2.2.2. Radiologic Data: CT Imaging Protocol

CT was performed using a 4- and 16-channel multidetector CT scanner (Mx 8000; Marconi Medical Systems, Tel Aviv, Israel; and LightSpeed Pro 16, GE Healthcare, Milwaukee, WI, USA). The images were captured in a craniocaudal direction. The CT was performed before and after contrast injection. The patients were administered intravenous contrast material (iopromide, Ultravist 300; Schering, Berlin, Germany) via the antecubital vein, using a mechanical injector (120 mL at 3 mL/s). Scanning for early-phase images began 35 s after the intravenous contrast injection, from the neck to the upper abdomen.

#### 2.2.3. Radiologic Data: Evaluation of the Hounsfield Units

All of the images were assessed using a picture archiving and communication system (Centricity Radiology RA 1000; GE Healthcare, Barrington, IL, USA) that displayed the image data on the monitors. Two independent personnel (S Han; KM Kim) who were blinded to the study analyzed the HU as part of the study protocol. The maximum HU of the regions of interest (ROIs) were obtained from three measurements taken on the corresponding post-contrast image by each individual. A manually controlled ROI was placed in the main lesion of the breast. Briefly, a circle of ≥2 mm was drawn on the mass, that covered >50% of the mass while excluding peripheral areas to avoid partial volume effects from the adjacent normal parenchyma. The calcified lesions, blood vessels, and necrotic and/or cystic areas were excluded from the ROI measurements. The maximum HU of the tumor-to-aorta ratio (TAR) was calculated as the ratio between the maximum tumor and the aortic arch HU values from the contrast-enhanced images.

### 2.3. Statistical Analysis

Descriptive statistics were used to describe the patients’ baseline characteristics. For the comparison of the two groups, the chi-square test or Fisher’s exact test were used for applicable categorical values. An independent *t*-test was used to continuously compare the two values.

Performance capacity as a prognostic factor was evaluated under the auspices of the Youden’s index, which served to determine the appropriate cut-off values from the area under the receiver operating characteristic curve (AUROC). The primary endpoints were the recurrence-free interval (RFI) and overall survival (OS). In accordance with the standardized definitions for efficacy endpoints in the adjuvant breast cancer trial criteria [16], the RFI was defined as the time from the date of primary diagnosis to the date of one of the following events: invasive ipsilateral breast tumor recurrence; local or regional invasive recurrence; distant recurrence; or death from breast cancer. OS was defined as the time from the date of primary diagnosis to the date of death from any cause of breast cancer, non-breast cancer, or an unknown cause [16]. Kaplan–Meier (KM) curves were generated, and the log-rank test was used to compare survival between the different groups. The RFI and OS at 12 months were calculated using KM curves. A Cox proportional hazards model was used to estimate the hazard ratios with 95% confidence intervals for the univariate and multivariate approaches. The subgroup analysis with the TAR, NLR, and their combination was planned, based on recent studies using the following groups: low TAR and low NLR; low TAR and high NLR; high TAR and low NLR; and high TAR and high NLR, [6,11,12,13,17,18,19]. Further analysis in a two-group manner was planned to determine if there was any survival difference that could dichotomize the patients into two groups. All of the statistical analyses were performed using IBM SPSS Statistics version 25 (IBM Corp., Armonk, NY, USA). All of the *p*-values were two-sided, and the statistical significance level was set at *p* < 0.05.

## 3. Results

### 3.1. Clinicopathologic Findings

**Figure 1:**
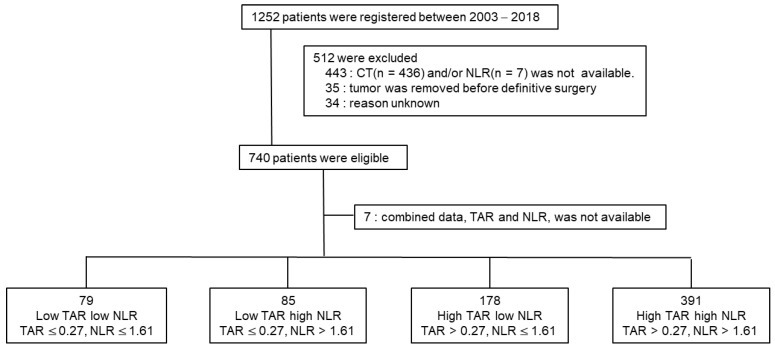
Study profile. A total of 1252 patients were diagnosed with primary breast cancer and completed their planned treatment between 2003 and 2018. Data of 740 patients were analyzed after excluding 512 patients who did not meet the eligibility criteria due to computed tomography (CT) not taken preoperatively (*n* = 406), CT images were not archived (*n* = 37), the tumor was removed before definitive surgery (*n* = 35), and unknown reason (*n* = 34). Among the 740 patients, 733 patients had both radiologic and serologic data to analyze the Hounsfield units of the tumor-to-aorta and neutrophil-to-lymphocyte ratios.

In total, 740 patients were eligible for this study (Figure 1).

The mean age of the patients was 54.3 ± 11.3 (range, 22–89) years. The mean tumor size was 2.49 ± 1.79 (range, 0.1–15.0) cm, and approximately half of the patients had T1 disease (46.4%). Two-thirds of the patients had node-negative disease (63.1%), and the average number of nodal metastasis was 1.5 ± 4.6 (range, 0–36). Two-thirds of the patients had hormone receptor-positive disease (464/740), and a quarter had HER2 over-expressed disease (189/740). The detailed patient demographics are described in Table 1.

### 3.2. RFI Disadvantage of TAR > 0.27 and NLR > 1.61

The mean HU values of the tumor and aorta on contrast-enhanced CT were 115.68 ± 32.60 (range, 18.0–283.0) and 338.62 ± 50.91 (range, 204.0–602.0), respectively (Table 2). The TAR of HU was 0.348 ± 0.108 (range, 0.062–1.11) (Table 2).

**Figure 2:**
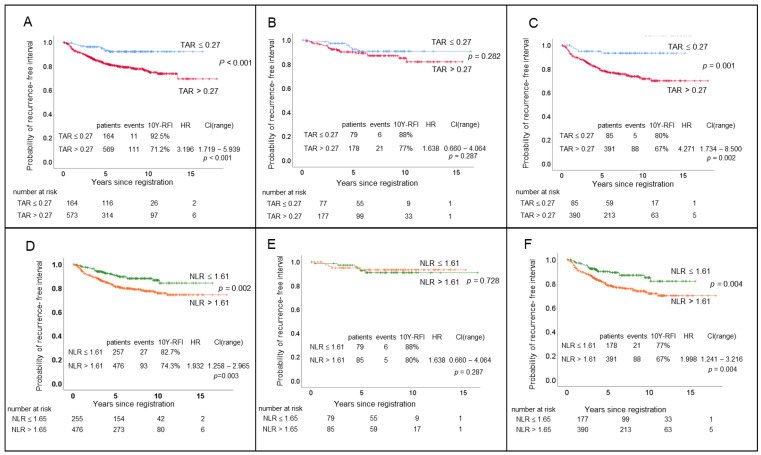
The recurrence-free interval (RFI) among patients with high and low tumor-to-aorta ratios (TAR) of Hounsfield units on contrast-enhanced computed tomography and serum neutrophil-to-lymphocyte ratio (NLR). Patients were dichotomized into high and low TAR groups with a cut-off value of 0.27 (**A**–**C**) and high and low NLR groups with a cut-off value of 1.61 (**D**–**F**). The RFI was defined as the time from pathological diagnosis of primary disease to invasive ipsilateral breast tumor recurrence, local/regional invasive recurrence, distant recurrence, or death from breast cancer, whichever occurred first. A Kaplan–Meier (KM) graph was generated and compared with a log-rank test to see the RFI difference. Patients with high TAR (**A**) and high NLR (**D**) showed worse RFI than their counterparts. However, this RFI disadvantage of high TAR and high NLR was found only when patients had high values of both TAR and NLR (**C**,**F**). Y, year; HR, hazard ratio; CI, confidence interval.

The performance capacity of TAR as a prognostic factor in the RFI and OS with AUROC was 0.602 and 0.638, respectively. The patients were divided into high and low TAR groups, with a cut-off value of 0.27 in the RFI and 0.37 in OS (Table S1A,B, Supplementary Materials). The rate of RFI at ten years among the patients with a TAR > 0.27 was 71.2%, as compared with 92.5% in patients with a TAR ≤ 0.27, with a significant survival disadvantage (hazard ratio (HR), 3.19; confidence interval (CI), 1.719–2.939; *p* < 0.001) (Figure 2A). The disadvantage of the RFI existed only in the patients with an NLR > 1.61. The patients with an NLR ≤ 1.61 showed no difference in the RFI regardless of the TAR (*p* = 0.282) (Figure 2B). In contrast, in the patients with an NLR > 1.61, the TAR was a significant prognostic factor (HR, 4.271; CI, 1.734–10.519; *p* = 0.002), and the RFI was significantly different between the high and low TAR groups (*p* = 0.001) (Figure 2C). A similar pattern of survival disadvantage of the TAR was observed when the OS difference was explored (Figure S1A–C, Supplementary Materials).

The mean serum neutrophil and lymphocyte counts were 3.90 ± 1.66 (0.73–13.50) E9/L and 1.94 ± 0.69 (0.33–4.38) E9/L, respectively. The NLR was 2.29 ± 1.60 (0.61–20.30) (Table 2). The performance of the NLR as a prognostic factor was examined using AUROC. The associated RFI and OS were reported as 0.559 and 0.573, respectively. The patients were divided into high and low NLR groups, with a cut-off value of 1.61 in the RFI and 1.65 in OS (Table S1C,D, Supplementary Materials). The rate of RFI at ten years was 74.3% for the patients with an NLR > 1.61, which was significantly lower than that in the patients with an NLR < 1.61, where the rate of RFI at ten years was 82.7% (HR, 1.932; CI, 1.258–2.969; *p* = 0.003) (Figure 2D). However, this survival disadvantage was observed only in the patients with a TAR higher than 0.27 (HR 1.998; CI, 1.241–3.216; *p* = 0.004) (Figure 2F), and the patients with a low TAR did not show a different RFI, regardless of their NLR value (*p* = 0.728) (Figure 2E,F). A similar pattern of survival disadvantage of the NLR was observed when OS was explored (Figure S1D–F, Supplementary Materials).

### 3.3. TAR and NLR as Independent Risk Factors

**Figure 3:**
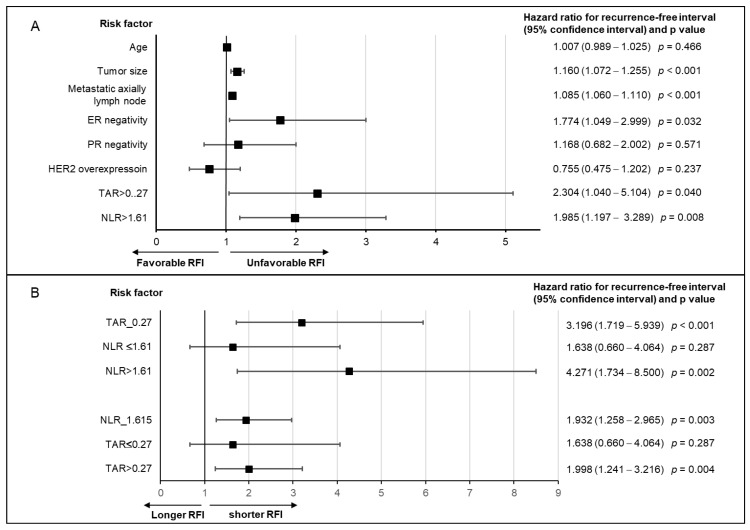
Effect of clinical factors, the tumor-to-aorta ratio of Hounsfield units on contrast-enhanced computed tomography (TAR), and the serum neutrophil-to-lymphocyte ratio (NLR) on recurrence-free interval (RFI).(**A**) Forest plot A shows the hazard ratios (HRs) and 95% confidence intervals (CI) of age, tumor size, metastatic axillary lymph node, estrogen receptor (ER) negativity, progesterone receptor (PR) negativity, human epidermal receptor 2 (HER2) overexpression, TAR > 0.27, and NLR > 1.61 of RFI. The multivariate analysis with a Cox proportional hazard model showed that age, tumor size, the existence of metastatic disease at the axillary lymph nodes, ER negativity, TAR > 0.27, and NLR > 1.61 were independent risk factors for RFI. (**B**) Forest plot B indicates that patients with high TAR > 0.27 and NLR > 1.61 had a significant disadvantage of RFI only when they had a high value of the other factor.

Both the TAR and NLR were independent risk factors for the RFI, along with the tumor size, the presence of metastatic disease at the axillary lymph node, and negative ER (Figure 3A). Subgroup analysis revealed that high TAR and NLR showed a significant survival disadvantage only when the other factor was also higher than the cut-off value (Figure 3B). A similar pattern of significance as a prognostic factor was observed when high TAR and high NLR were evaluated in the context of OS (Figure S2A,B, Supplementary Materials).

### 3.4. Combined TAR and NLR Produced a Significant Independent Prognostic Factor, High-High

**Figure 4:**
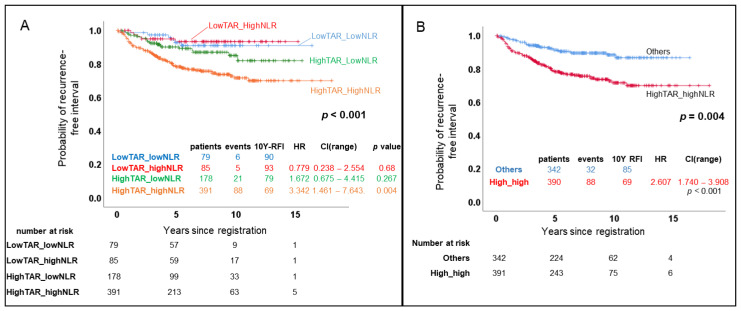
Recurrence-free interval (RFI) of patients categorized into four groups according to the high and low values of the tumor-to-aorta ratio (TAR) of Hounsfield units on contrast-enhanced computed tomography and serum neutrophil-to-lymphocyte ratio (NLR). (**A**) A Kaplan–Meier (KM) graph was generated and compared with a log-rank test. This showed that only patients with high TAR and high NLR showed worse prognoses than other groups; (**B**) Patients were dichotomized into two groups: those with high values of both TAR and NLR (high-high) and the other patients with only one or fewer factors higher than the cut-off value (the others). The high-high group had a significantly worse prognosis than the other groups.

The rate of RFI at ten years was 69% when the patients had both a high TAR and a high NLR, as compared with 90% and 93% when the patients had a low TAR regardless of the NLR, and 79% when the patients had a high TAR but a low NLR (HR, 3.342; CI, 1.461–7.643; *p* = 0.004) (Figure 4A). Further analysis was undertaken after dichotomizing the patients into two groups, patients with a TAR > 0.27 and an NLR > 1.61 defined as ‘high-high’, and the other three groups defined as ‘the others’. The patients in the high-high group were younger and had larger and more ER-negative tumors. The detailed profiles are listed in Table 3.

**Figure 5:**
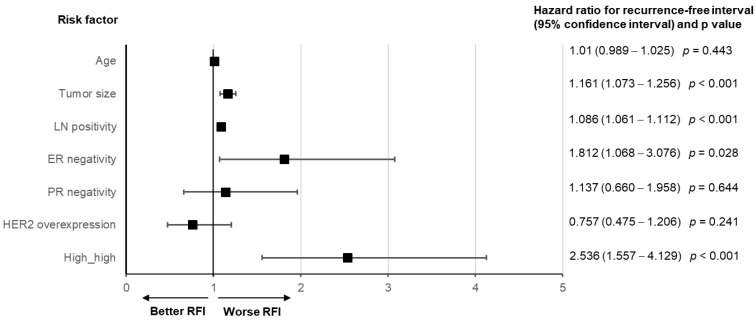
Effect of clinical factors and the combined factor of the tumor-to-aorta ratio (TAR) of Hounsfield units on contrast-enhanced computed tomography larger than 0.27 and the serum neutrophil-to-lymphocyte ratio (NLR) larger than 1.61, represented as ‘high-high’ on recurrence-free interval (RFI). Multivariate analysis with Cox’s proportional hazard model showed that tumor size, metastatic axillary lymph node disease, negativity of estrogen receptors, and high-high were significant independent prognostic factors. The hazard ratios and confidence intervals are provided on the left side of the plot.

The multivariate analysis revealed that high-high was an independent risk factor for the RFI (HR, 2.536; CI, 1.557–40,129; *p* < 0.001), tumor size, metastatic disease of the axillary lymph nodes, and negative ER (Figure 5). The combination of the TAR and produced a similar OS pattern (Figures S3 and S4, Supplementary Materials).

## 4. Discussion

We confirmed that the TAR and NLR had a reciprocal effect on patient survival (Figure 2; Figure S1, Supplementary Materials). Moreover, the patients showed significantly worse survival only when they had a high TAR and high NLR, suggesting a close relationship between the tumor vascularity and systemic immunity (Figure 4; Figure S3, Supplementary Materials).

Tumor vascularity is known to play a fundamental role in promoting cancer growth, invasion, and metastasis in various cancers, including breast cancer [3]. The HU on contrast-enhanced CT has been considered a feasible approach to evaluate tumor vascularity, because measuring CT intensity is non-invasive and can be standardized, which facilitates a comparison across multiple and repetitive imaging systems [20]. The concentration of iodine within the blood vessels during the intravenous administration of conventional iodinated contrast material is linearly proportional to the resultant increase in attenuation [21,22]. Therefore, the intensity data of the tumors on contrast-enhanced CT can be translated as a surrogate marker, indicating the vascular support of the tumor [21,22]. We confirmed that the TAR applied as a biomarker reflecting tumor vascularity showed significant capacity as a prognostic factor and had an impact on the patients’ RFI and OS (Figure 2; Figure S1, Supplementary Materials). This study has shown that, by utilizing reference values to reduce inter-observer variability, in this case, HU on the aortic arch, the TAR could become more practical to apply in clinical practice.

The NLR is known to be related to patient survival [8,9,23], and we also confirmed results consistent with those of previous studies (Figure 2; Figure S1, Supplementary Materials). Moreover, after a significant association between the NLR and immune checkpoint inhibitors (ICIs) was reported [7], the NLR was proposed as a promising biomarker for ICIs, because there is no systemic immune biomarker that can be widely used to guide treatment for the patients with early breast cancer with immune checkpoint inhibitors [7,24,25].

Indeed, no vascular or immune biomarker has been sufficiently established to permit bedside decision-making to direct ICIs or anti-angiogenic agents for early breast cancer [7,11,24,25,26,27]. For example, randomized phase III trials with pembrolizumab (KEYNOTE-522 ClinicalTrials.gov number, NCT03036488) and atezolizumab (Impassion031 ClinicalTrials.gov number, NCT03197935) showed the consistent benefits of immunotherapy in early triple-negative breast cancer across subgroups, with or without biomarker expression and programmed death receptor 1 or its ligand positivity, respectively [24,25]. There is no biomarker for anti-angiogenic agents, such as bevacizumab, to identify the patients who would benefit from these agents [27]. These findings, and recent reports regarding the crosstalk between vascularity and immunity, could promote the further exploration of the crosstalk between vascularity and immunity in patients with breast cancer.

The bidirectional relationship between tumor vascularity and host immunity has attracted the attention of basic and translational researchers. Tumor vasculature has been reported as a key component of the microenvironment that can influence the immune cells in the circulation, because structural and functional abnormalities of tumor vasculature, as well as excessive angiogenesis, set up difficult hurdles for leukocyte recruitment, which eventually alters the systemic immune response [11,22]. Meanwhile, there is also increasing evidence that systemic immune components play a key role in the induction of angiogenesis in cancer [13]. The preclinical and clinical studies have shown markedly less vascularization of tumors when the host is in a disturbed state of monocytes or macrophages [13]. These cells are known to function as vascular modulators by producing a variety of proangiogenic factors [13,28]. The reciprocal effect between tumor vascularity and systemic immunity was confirmed in this study, utilizing TAR as a representative factor of tumor vascularity and the NLR as a representative factor of systemic immunity (Figure 2 and Figure 3; Figures S1 and S2, Supplementary Materials). Remarkably, this study proved the concept of crosstalk between vascularity and host immunity in the context of clinical features. To the best of our knowledge, this is the first study to report a reciprocal effect between tumor vascularity and systemic immunity in patients. Although the TAR and NLR were independent risk factors, they also showed complementary features. Further understanding of the compatibility between tumor vascularity and host immunity would help utilize agents against them in the clinic.

The limitations of this study were mostly due to its retrospective nature. Of the 1252 patients, 443 (35.4%) did not undergo CT. The national comprehensive cancer network (NCCN) guideline, and the institution guideline implemented the NCCN guideline, do not recommend CT unless patients have suspicious symptoms suggestive of distant metastasis [29]. Patients with a high risk for distant metastasis were inevitably included in this policy, so more than half of the patients were categorized under the high-risk group. Despite this, the results of this study provided meaningful data for patients with a high risk of recurrent disease, who urgently require biomarkers for tumor vascularity and host immunity. Another limitation is that the systemic treatment was not controlled, which is inevitable in a retrospective study of patients treated in daily practice. Further validation is required in other cohorts, and prospective randomized trials should be conducted. Finally, different cut-off values were used for the RFI and OS, as exploring the exact number of cut-off values was outside the scope of the current study. A particular value, such as “greater than 25” in the 21-gene Recurrence Score assay, which indicates that patients with an estrogen receptor–positive breast cancer would benefit from chemotherapy, or a range of values, such as 1 to 25 in the same assay, indicating who can safely forego chemotherapy, would be more practicable in the clinical field [30,31]. Further investigations in the basic and clinical fields will hopefully yield certain values of TAR and NLR for clinical utility.

## 5. Conclusions

The TAR and NLR showed a reciprocal influence on patient survival, and factors from a combination of these two factors had a significant impact on patient survival, indicating potential capacity as a feasible biomarker in the clinic. Understanding the biological background behind these clinical features is urgent, for the further application of these two factors, TAR and NLR, in clinical practice. Thus, we hope that this retrospective clinical research can promote further research to elucidate the crosstalk between tumor vascularity and host immunity.

## Figures and Tables

**Table 1:** Clinicopathologic characteristics of the patients.

Variables	Subgroup	*N* = 740
* Age (years)		54.3 ± 11.3 (25–89)
* Tumor size(cm)		2.5 ± 1.8 (0.1–15.0)
T stage		
	T1	343 (46.4)
	T2	276 (37.3)
	T3	46 (6.2)
	Unknown	75 (10.1)
* Positive N		1.5 ± 4.6 (0–36)
N stage		
	N0	467 (63.1)
	N1	145 (19.6)
	N2	60 (8.1)
	N3	35 (4.7)
	Unknown	33 (4.5)
ER		
	Positive	464 (62.7)
	Negative	247 (33.4)
	Unknown	29 (3.9)
	Total	740 (100)
PR		
	Positive	393 (53.1)
	Negative	318 (43.0)
	Unknown	29 (3.9)
	Total	740 (100)
HER2		
	Negative	519 (70.1)
	Positive	189 (25.5)
	Unknown	32 (4.4)
	Total	740 (100)

Categorical values were described as a “number (%)” Continuous values with an asterisk (*) were described as the “mean ± standard deviation(minimum-maximum)”. T, tumor size; N, axillary lymph node; ER, estrogen receptor; PR, progesterone receptor; HER2, human epidermal growth factor receptor 2.

**Table 2:** Basic characteristics of Hounsfield units on computed tomography and serum neutrophil and lymphocyte counts.

Variables	Mean SD (Minimum–Maximum)
Pre-contrast HU	55.37 ± 23.60 (14.0–211.0)
Post-contrast HU	115.68 ± 32.60 (18.0–283.0)
Aorta HU	338.62 ± 50.91 (204.0–602.0)
TAR	0.348 ± 0.108 (0.062–1.11)
Neutrophil (E9/L)	3.90 ± 1.66 (0.73–13.50)
Lymphocyte (E9/L)	1.94 ± 0.70 (0.33–4.38)
NLR	2.29 ± 1.53 (0.61–10.47)

SD, standard deviation; HU, Hounsfield units; TAR, tumor-to-aortic arch ratio of HU; NLR, neutrophil-to-lymphocyte ratio.

**Table 3:** Clinicopathologic characteristics of two groups, high-high and the others.

Variables		High-High	Non-High-High	*p*-Value
Age		51.96 ± 11.54	56.96 ± 10.67	<0.001
Tumor size		2.76 ± 2.11	2.25 ± 1.41	<0.001
Metastases to LN		2.12 ± 5.14	1.37 ± 3.74	0.03
ER				0.018
	Positive	230	231	
	Negative	146	100	
PR				0.172
	Positive	199	193	
	Negative	177	138	
HER2				0.202
	Negative	266	249	
	Positive	108	81	

High-high, patients with TAR > 0.27 and NLR > 1.61; LN, lymph node; ER, estrogen receptor; PR, progesterone receptor; HER2, human epidermal growth factor receptor 2. The high-high group showed a significantly worse RFI than the others group (HR, 2.607; CI, 1.740–3.908; *p* < 0.001) (Figure 4B).

## Data Availability

The data presented in this study are available on request from the corresponding author. The data are not publicly available due to the personal information protection act in Korea.

## Supplementary Materials

The following supporting information can be downloaded at: https://www.mdpi.com/article/10.3390/cancers14143322/s1, Figure S1. Hounsfield units analyses on pre-and post-contrast enhanced computed tomography in a 63-year old woman with invasive ductal cancer of the left breast; Figure S2: Overall survival (OS) among patients with high and low tumor-to-aorta ratios (TAR) of Hounsfield units on contrast-enhanced computed tomography and the serum neutrophil-to-lymphocyte ratio (NLR); Figure S3: Effect of clinical factors, the tumor-to-aorta ratio of Hounsfield units on contrast-enhanced computed tomography (TAR), and the serum neutrophil-to-lymphocyte ratio (NLR) on overall survival (OS); Figure S4: Overall survival (OS) of patients categorized into four groups according to the high and low values of the tumor-to-aorta ratio (TAR) of Hounsfield units on contrast-enhanced computed tomography and the serum neutrophil-to-lymphocyte ratio (NLR); Figure S5: Effect of clinical factors and the combined factor of the tumor-to-aorta ratio (TAR) of Hounsfield units on contrast-enhanced computed tomography larger than 0.37 and the serum neutrophil-to-lymphocyte ratio (NLR) larger than 1.65, represented as ‘high-high’ on overall survival (OS); Table S1: Coordinates of the receiver operating characteristic curve.

## Abbreviations

ASCO/CAP	the American Society of Clinical Oncology/College of American Pathologists
AUROC	the area under the receiver operating characteristic curve
CT	computed tomography
ER	estrogen receptor
HER2	Human epidermal receptor 2
HU	Hounsfield units
KM	Kaplan–Meier
LN	lymph node
NLR	neutrophil to lymphocyte ratio
OS	overall survival
PR	progesterone receptor
RFI	recurrence-free interval
ROIs	the regions of interest
TAR	tumor-to-aorta ratio of HUs on contrast enhanced CT scan
