# Supplementary material for: Usefulness of Hounsfield Units and the Serum Neutrophil-to-Lymphocyte Ratio as Prognostic Factors in Patients with Breast Cancer
Source: Cancers (Basel). 2022 Jul 7;14(14):3322. doi: 10.3390/cancers14143322 (PMC9318691; doi:10.3390/cancers14143322)
Supplement: Supplementary file 1 [file cancers-14-03322-s001.zip › cancers-1788460-supplementary.pdf]

# Supplementary figures

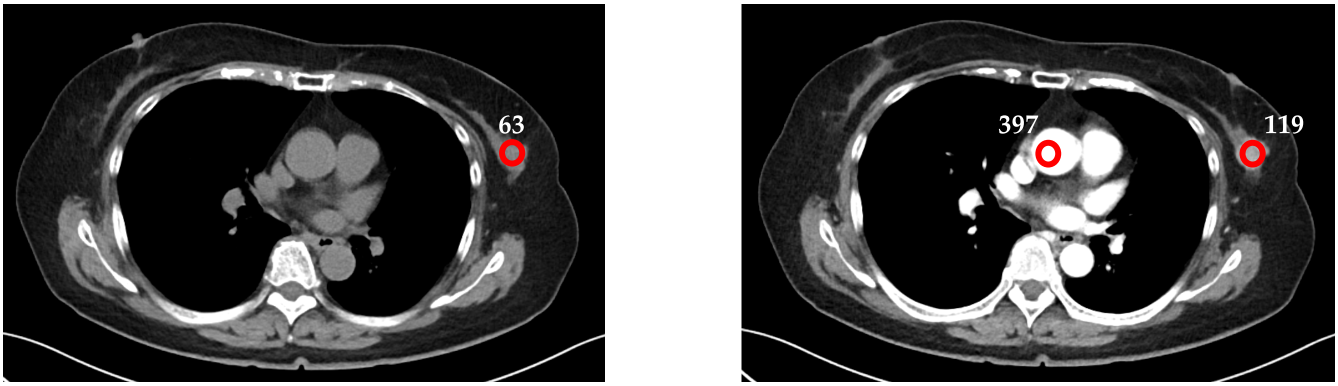


Supplementary Figure S1. Hounsfield units analyses on pre-and post-contrast enhanced computed tomography in a 63-year old woman with invasive ductal cancer of the left breast . (A) Axial CT image shows a mass in left breast. Region of interest is set on the mass and HU was measured as 63 (B) Same cut after contrast enhancement shows a mass in the left breast , which was enhanced with contrast. In the same cut, aortic arch was also measured.

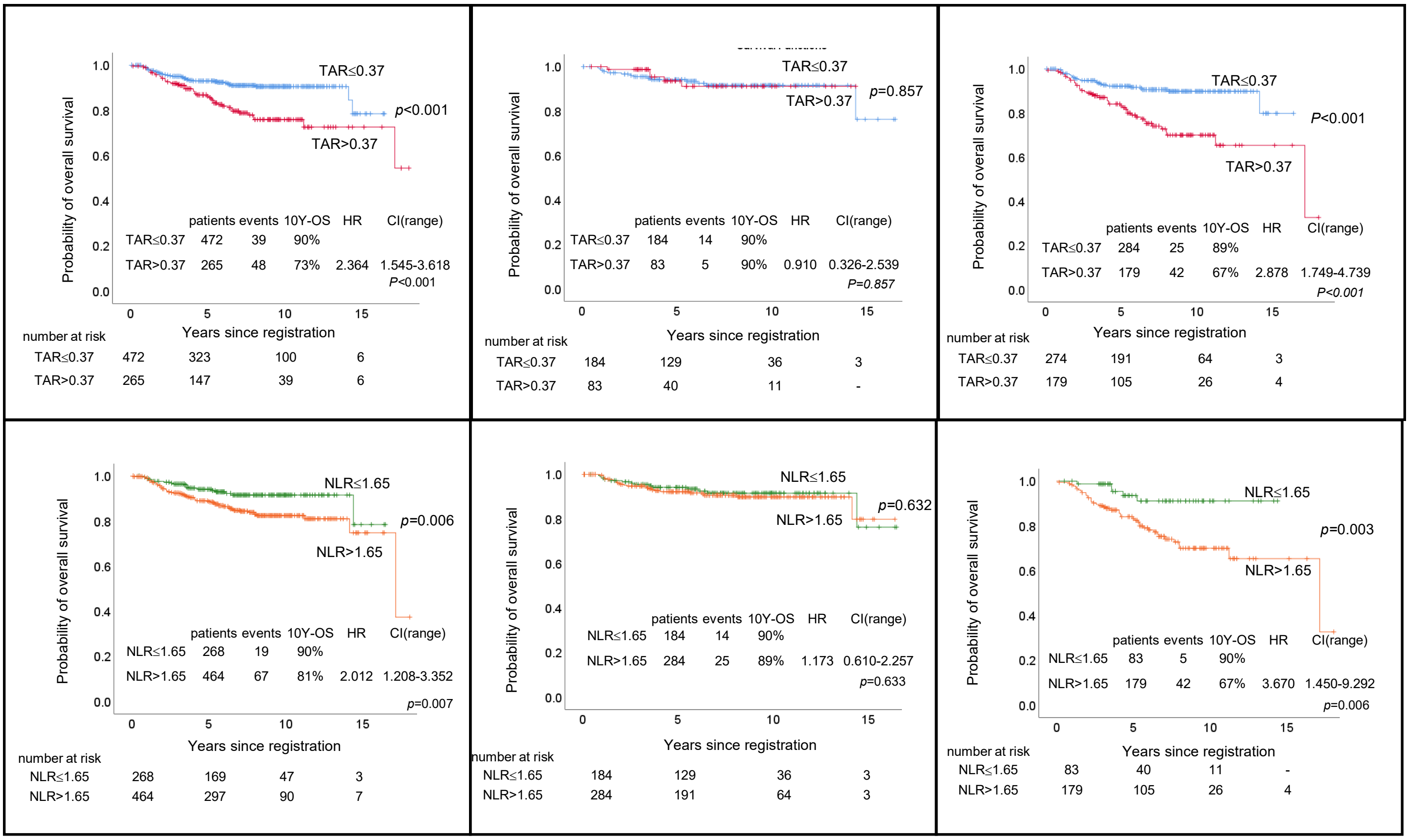


Supplementary Figure S2. The overall survival (OS) among patients with high and low tumor-to-aorta ratios (TAR) of Hounsfield units on contrast-enhanced computed tomography and serum neutrophil-to-lymphocyte ratio (NLR). Patients were dichotomized into high and low TAR groups with a cut-off value of 0.37 (A, B, and C) and high and low NLR groups with a cut-off value of 1.65 (D, E, and F). The OS was defined as the time from pathological diagnosis of primary disease to death from any cause. A Kaplan-Meier graph was generated and compared with a log-rank test to see the OS difference. Patients with high TAR (A) and high NLR (D) showed worse OS than their counterparts. However, this OS disadvantage of high TAR and high NLR was found only when patients had high values of both TAR and NLR (C and F). Y, year; HR, hazard ratio; CI, confidence interval.

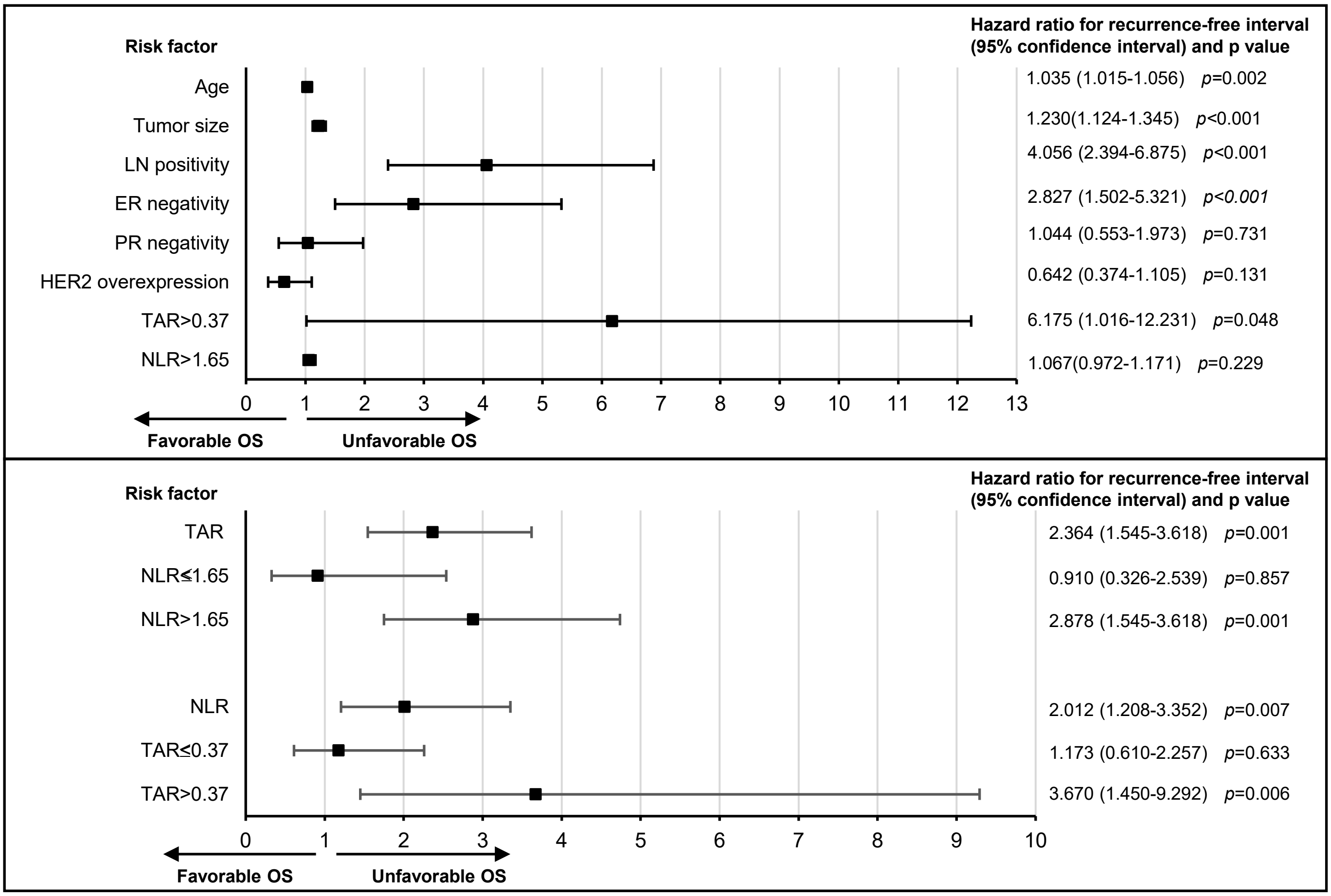


Supplementary Figure S3. Effect of clinical factors, the tumor-to-aorta ratio of Hounsfield units on contrast-enhanced computed tomography (TAR), and the serum neutrophil-to-lymphocyte ratio (NLR) on overall survival (OS). Forest plot A shows the hazard ratios (HRs) and 95% confidence intervals (CI) of age, tumor size, metastatic axillary lymph node, estrogen receptor (ER) negativity, progesterone receptor (PR) negativity, human epidermal receptor 2 (HER2) overexpression, TAR>0.37 and NLR>1.65 of OS. The multivariate analysis with a Cox proportional hazard model showed that age, tumor size, the existence of metastatic disease at the axillary lymph nodes, ER negativity, TAR>0.37, and NLR>1.65 were independent risk factors for OS. Forest plot B indicates that patients with high TAR>0.37 and NLR>1.65 had a significant disadvantage of OS only when they had a high value of the other factor.

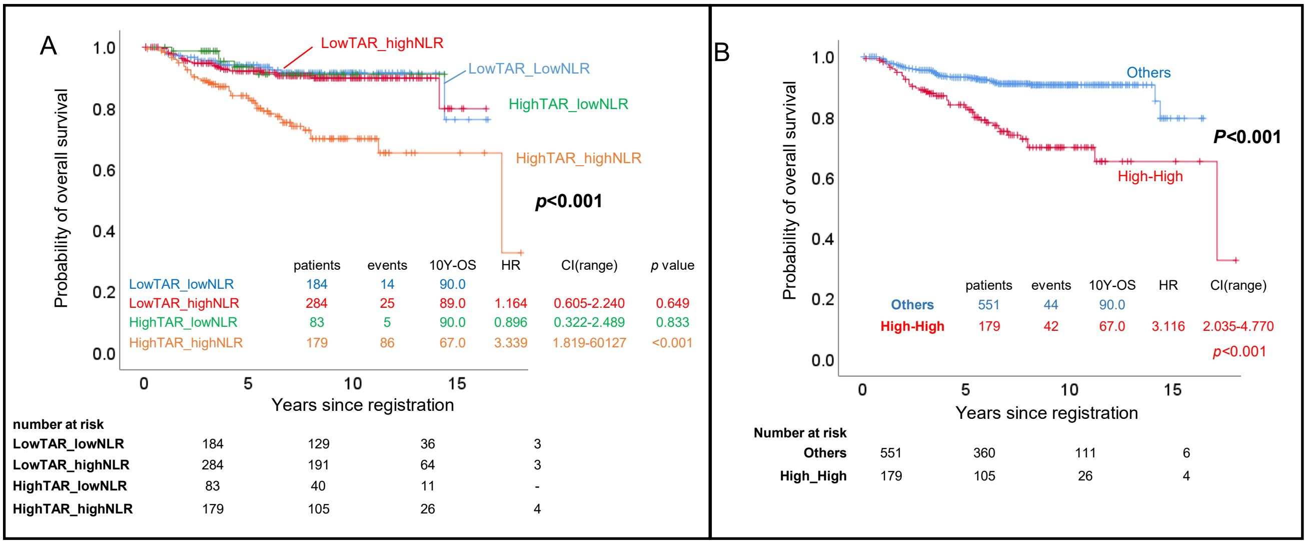


Supplementary Figure S4. Overall survival(OS) of patients categorized into four groups according to the high and low values of the tumor-to-aorta ratio (TAR) of Hounsfield units on contrast-enhanced computed tomography and serum neutrophil-to-lymphocyte ratio (NLR). A Kaplan-Meier graph was generated and compared with a log-rank test. (A) Patients with high TAR and high NLR showed worse prognoses than other groups. (B) Patients were dichotomized into two groups: those with high values of both TAR and NLR (high-high) and the other patients with only one or fewer factors higher than the cut-off value (others). The group “high-high” had a significantly worse prognosis than group “Others.”

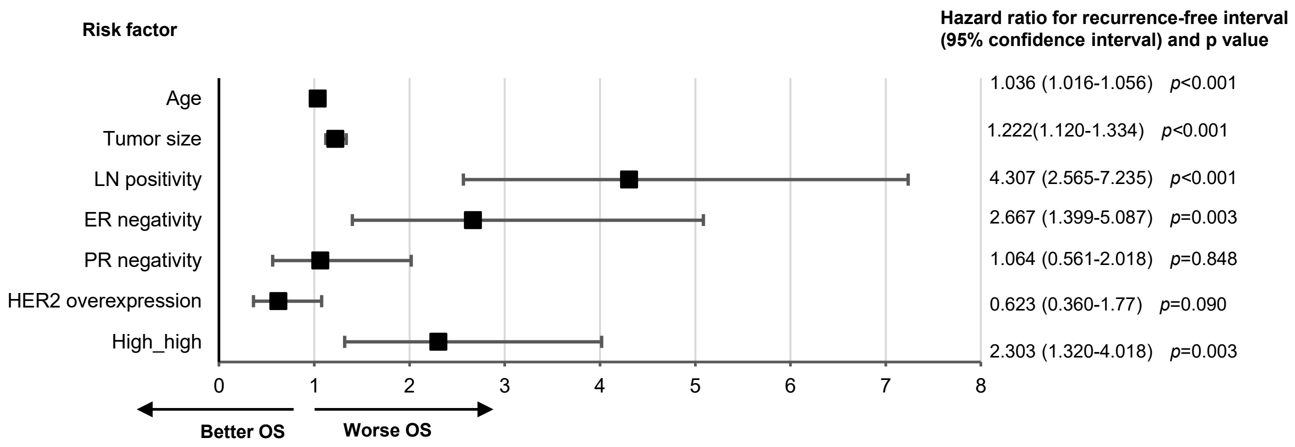


Supplementary Figure S5. Effect of clinical factors and the combined factor of the tumor-to-aorta ratio (TAR) of Hounsfield units on contrast-enhanced computed tomography larger than 0.37 and the serum neutrophil-to-lymphocyte ratio (NLR) larger than 1.65, represented as 'High-High' on overall survival (OS). Multivariate analysis with Cox's proportional hazard model showed that patients' age, tumor size, metastatic axillary lymph node disease, negativity of estrogen receptors, and high-high were significant independent prognostic factors. The hazard ratios and confidence intervals are provided on the left side of the plot.
